# The role and uses of antibodies in COVID-19 infections: a living review

**DOI:** 10.1093/oxfimm/iqab003

**Published:** 2021-01-28

**Authors:** D Oliver Scourfield, Sophie G Reed, Max Quastel, Jennifer Alderson, Valentina M T Bart, Alicia Teijeira Crespo, Ruth Jones, Ellie Pring, Felix Clemens Richter, The Oxford-Cardiff COVID-19 Literature Consortium, David J Ahern, Hannah Almuttaqi, Dominic S Alonzi, Aljawharah Alrubayyi, Ghada Alsaleh, Valentina M T Bart, Vicky Batchelor, Rebecca Bayliss, Dorothée L Berthold, Jelena S Bezbradica, Tehmina Bharuchq, Helene Borrmann, Mariana Borsa, Rowie Borst, Juliane Brun, Stephanie E A Burnell, Lorenzo Capitani, Athena Cavounidis, Lucy Chapman, Anne Chauveau, Liliana Cifuentes, Amy Susan Codd, Ewoud Bernardus Compeer, Clarissa Coveney, Amy Cross, Sara Danielli, Luke C Davies, Calliope A Dendrou, Sandra Dimonte, Ruban Rex Peter Durairaj, Lynn B Dustin, Arthur Dyer, Ceri Fielding, Fabian Fischer, Awen Gallimore, Sarah Galloway, Anís Gammage, Ester Gea-Mallorquí, Andrew Godkin, Stephanie Jean Hanna, Cornelia Heuberger, Sarah Hulin-Curtis, Fadi Issa, Emma Jones, Ruth Jones, Kristin Ladell, Sarah N Lauder, Kate Liddiard, Petros Ligoxygakis, Fangfang Lu, Bruce MacLachlan, Shayda Maleki-Toyserkani, Elizabeth H Mann, Anna M Marzeda, Reginald James Matthews, Julie M Mazet, Anita Milicic, Emma Mitchell, Owen Moon, Van Dien Nguyen, Miriam O'Hanlon, Clara Eléonore Pavillet, Dimitra Peppa, Ana Pires, Eleanor Pring, Max Quastel, Sophie Reed, Jan Rehwinkel, Niamh Richmond, Felix Clemens Richter, Alice J B Robinson, Patrícia R S Rodrigues, Pragati Sabberwal, Arvind Sami, Raphael Sanches Peres, Quentin Sattentau, Barbora Schonfeldova, David Oliver Scourfield, Tharini A Selvakumar, Freya R Shepherd, Cariad Shorten, Anna Katharina Simon, Adrian L Smith, Alicia Teijeira Crespo, Michael Tellier, Emily Thornton, Lion F K Uhl, Erinke van Grinsven, Angus K T Wann, Richard Williams, Joseph D Wilson, Dingxi Zhou, Zihan Zhu, Stephanie E A Burnell

**Affiliations:** 1 Division of Infection and Immunity, School of Medicine, Cardiff University, Cardiff, CF14 4XN, UK; 2 Nuffield Department of Medicine, University of Oxford, Oxford, OX3 7FZ, UK; 3 Kennedy Institute of Rheumatology, NDORMS, University of Oxford, Oxford, OX3 FTY, UK; 4 Division of Cancer and Genetics, School of Medicine, Cardiff University, Cardiff, CF14 4XN UK; 5 Dementia Research Institute, Cardiff University, Cardiff, CF24 4HQ, UK

**Keywords:** antibodies, COVID-19, SARS-CoV-2, convalescent plasma, nanobodies, vaccines, long-term immunity

## Abstract

Coronavirus disease 2019 has generated a rapidly evolving field of research, with the global scientific community striving for solutions to the current pandemic. Characterizing humoral responses towards SARS-CoV-2, as well as closely related strains, will help determine whether antibodies are central to infection control, and aid the design of therapeutics and vaccine candidates. This review outlines the major aspects of SARS-CoV-2-specific antibody research to date, with a focus on the various prophylactic and therapeutic uses of antibodies to alleviate disease in addition to the potential of cross-reactive therapies and the implications of long-term immunity.

## INTRODUCTION

Humoral immunity is a vital aspect of the immune system highly implicated in infection control. Severe acute respiratory syndrome coronavirus 2 (SARS-CoV-2) is a highly infectious virus that is responsible for the current worldwide coronavirus disease 2019 (COVID-19) pandemic. Understanding the immune response to this virus is paramount to limit disease burden in the population, and to discover new therapeutic options. One such response is that of antibodies; the immunoglobulins secreted by B-cells following antigen recognition. Antibodies have a multitude of effector functions and can coordinate the responses of other immune cells, including T cells and macrophages, to eliminate pathogens. Studying the antibody response to SARS-CoV-2 will aid in vaccine design and the understanding of long-term immunity prospects. Additionally, antibodies that bind and neutralize the SARS-CoV-2 virus have the potential to be used as therapies for patients in the various forms of convalescent plasma, monoclonal antibodies and nanobodies, all of which are discussed within this review.

**Box 1:** What is the consensus on antibodies in SARS-CoV-2 infection?

When infected with COVID-19, patients produce antibodies to fight off the infection. These antibodies are known as immunoglobulins; IgM, IgA and IgG, and are key players in the response to COVID-19. Each has a unique role and therefore takes different lengths of time to be detected in the blood, to reach the maximum quantity and diminish from the system. As this is still a new disease, further work is needed to determine how long these antibody responses last in the body. Most COVID-19 patients that do not display any symptoms have low levels of IgM, while levels of IgA and IgG antibodies are higher in more severe, symptomatic patients. However, more in-depth study is needed to see if these antibody responses are important in controlling infection and how they co-ordinate with other immune responses to COVID-19.

Patients with strong immune responses to COVID-19 have high levels of neutralizing antibodies, which successfully control the infection. Once recovered, plasma can be taken from these patients and be administered to those who are currently severely infected. This is known as CP treatment. Other treatment options, which include mAbs and nanobodies, are more focused therapies, having developed from the most potent antibodies. Approval of two potent mAb therapies signifies the importance of antibodies in overcoming infection. However, these are most effective at preventing severe disease, so research to identify treatments to benefit those severely infected is still needed. However, these are most effective at preventing progression to severe disease, so research to identify treatments to benefit those severely infected is still needed. Work is also being carried out to investigate previous coronavirus infections to see what we can learn from them. It is possible that antibodies made against these other strains may help protect people during this pandemic.

It is currently unknown whether people who have recovered from COVID-19 are protected against a future SARS-CoV-2 infection as reinfection has been reported in several people worldwide. This has implications for vaccine design as regular boosters may be required if the immune response declines. Key components to creating a long-lasting immunity to the virus will become clearer once further research has been conducted.

**Box 2:** Why do antibodies in SARS-CoV-2 infection matter?

COVID-19 has rapidly changed the World, from countless deaths and long-term health problems in survivors, to creating a social and economic burden. Research on COVID-19 is being produced quickly, so it is crucial that we view this critically to distinguish robust data. From this baseline, we are then able to produce successful therapies as soon as possible to help fight this pandemic.

Looking at previous coronavirus strains is necessary to gain useful insights into this new and novel virus. There are similarities between SARS-CoV-2 and former strains we have faced, which give us invaluable knowledge in treating patients and limiting global disease burden. What we learn from COVID-19 may also be applied to future epidemic or pandemic strains.

Using antibodies taken from patients that have recovered from COVID-19 infection and giving them to those that are struggling to fight off the infection has the potential to save lives and bridge the gap while doctors and scientists are learning more about how to fight the virus and produce other treatments and vaccines.

## ANTIBODY RESPONSES TO SARS-CoV-2 IN DIFFERENT PATIENT POPULATIONS

**Figure 1:**
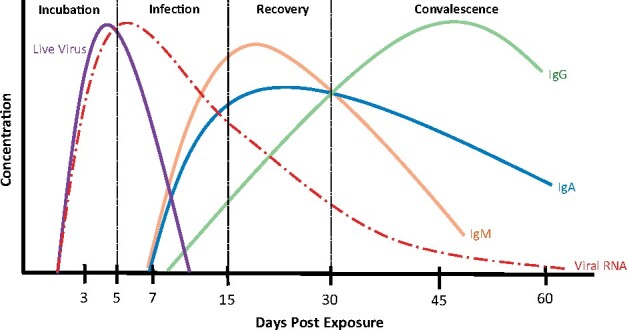
Changes in antibody concentration in response to viral infection. Following exposure to the virus and the initial incubation period of around 5 days, the infection takes hold. During the infection period, patients may develop symptoms as the first virus-specific antibodies are produced and the immune system is activated. IgM and IgA are produced initially, followed by IgG, which increases more slowly, but remains in the blood for a longer period. During the recovery and convalescent phases of infection, the viral RNA reduces to undetectable levels. IgA levels can persist, particularly at the mucous membranes [1–3], it is currently unclear how long the IgG titres last.

Immunoglobulins IgM, IgA and IgG are key components of the antibody response towards SARS-CoV-2 and differ in titre and duration of response, as with other viral infections (Figure 1) [4]. Table 1 summarizes the SARS-CoV-2 antibody literature to date. This includes seroconversion; how long it takes antibodies to be detected in the serum following infection, response kinetics; how long it takes antibodies to achieve their peak titre, and the prediction of response duration.

**Table 1:** Summary of analysis of IgM, IgA and IgG responses to SARS-CoV-2 infection

	IgM	IgA	IgG
Per cent seroconversion	>73 [5–9]	>72 [5, 6, 10]	84–100 [5–12]
Seroconversion (d.p.s.o)	10–14 [8, 13–16]	13 [16]	12–14 [8, 13–16]
Peak titre (d.p.s.o)	15–30 [3–5, 7, 9, 14, 17–19]	16–30 [3, 5, 20–23]	16–50 [3–7, 9, 17, 19, 20, 22–24]
Median seronegative prediction	46.9 days [6]	51.0 days [6]	

Following infection by SARS-CoV-2, IgM, IgA and IgG are rapidly seroconverted within the first 2 weeks; IgM and IgA appear to reach their peak titre at similar d.p.s.o, whereas IgG often peaks at a later time point.

IgG levels were shown to peak earlier in asymptomatic and mild cases compared to severe cases [∼20 vs. ∼35 days post symptom onset (d.p.s.o)] and most asymptomatic patients, many of whom were children, had low or undetectable IgM levels, leading to speculation that high and persistent IgM may result in more severe symptoms [19, 21, 25–27]. Interestingly, many publications have shown significant correlations of higher antibody titres in both older patients and those with more severe disease [7, 13, 17, 19, 28–30]. Relative levels of IgA and IgG have been reported to be significantly higher in severe patients in addition to a significant correlation between IgA levels and APACHE-II score in critically ill patients [16, 23]. A study investigating the specificity and functionality of antibody responses in children found that SARS-CoV-2 positive children had low levels of IgM, IgA and IgG when compared to severe COVID-19 adults and demonstrated that children predominantly generated an anti-S IgG response compared to the broader antibody response generated by adults [31]. It has been suggested that the reduced symptoms demonstrated by children could be due to the reduced expression of the viral receptor in children or that children generate a more robust innate immune response [32, 33].

In addition to age, biological sex is also a potential factor in COVID-19 disease severity. Several countries have reported higher hospital admissions and mortality rates in males, with a case fatality rate 1.7 times higher for men than for women [34]. The production of IgG appears to be higher in females in the early stages of infection, possibly preventing the progression to advanced disease and decreasing the mortality rate [35, 36]. Patients that succumb to SARS-CoV-2 infection were unable to generate a functional IgG response, coordinate Fc receptor-binding and produce innate immune effector binding [37]. Further to this, patients with severe COVID-19, particularly males, have been shown to generate IgG1 antibodies with significantly reduced Fc fucosylation, in addition to increased IgG3 antibodies when compared to patients with mild symptoms and children, indicating that severe COVID-19 resulted from the production of pro-inflammatory IgG antibodies [38].

Coordinated responses between B cells, CD4^+^ and CD8^+^ T cells are necessary to control and clear infection, without a functional B-cell response, virus-specific memory T cells cannot provide complete protection [39]. Neutralizing antibody (nAb) responses and B cell memory decline over time and depend on CD4^+^ T cell help, leaving the role of long-term protection to the memory T cells [40, 41]. This, therefore, indicates that the immune system as a whole must be analysed, in addition to the individual components, to understand why some people are asymptomatic while others succumb to the disease.

## THE USE OF ANTIBODIES AS THERAPY FOR COVID-19

**Figure 2:**
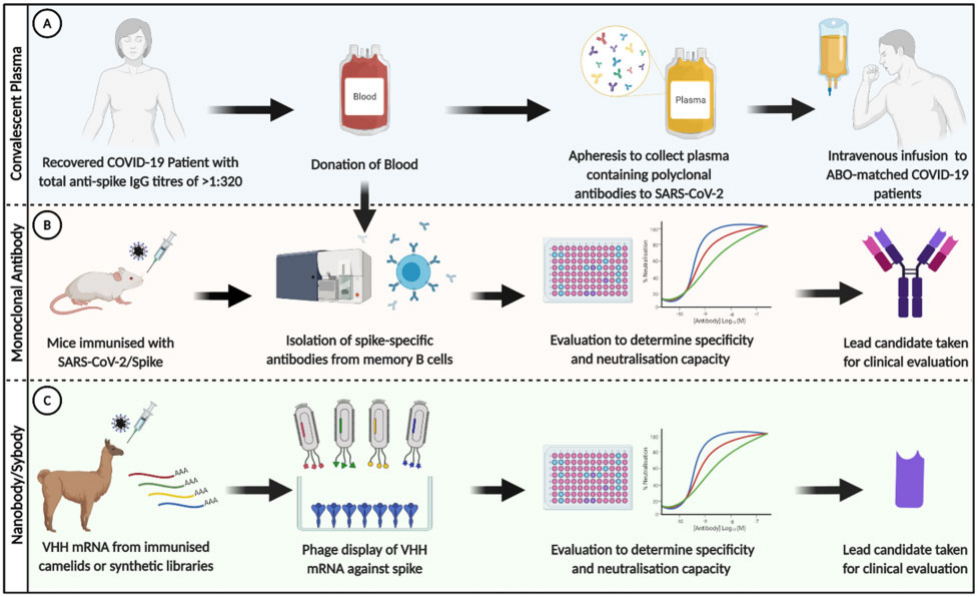
SARS-CoV-2-specific antibodies can be utilized in multiple ways to treat COVID-19. (**A**) Sera from recovered COVID-19 patients can be given intravenously as CP to ABO-matched-infected patients in order to reduce infectious burden and alleviate active disease. (**B**) mAbs (typically IgG) can be identified following isolation of spike/RBD-specific memory B cells, which are sourced from recovered patients or mice immunized with target antigen. Potential candidates are screened for various parameters, including specificity to target antigen and neutralization capacity. Selected lead candidates are further optimized before clinical evaluation as both a prophylactic and therapeutic. (**C**) The epitope-binding domain of antibody heavy chains (VHH) also has therapeutic potential. These can be isolated following immunization of camelids (nanobodies) or by using synthetic libraries (sybodies). The diversity of VHH mRNA is screened (e.g. using phage display) to identify those that have high affinity to the target antigen. Like mAbs, these are evaluated for their specificity and neutralization capacity before clinical evaluation. Figure created using BioRender.com

There are various strategies to treat SARS-CoV-2 infection with antibodies, as summarized in Figure 2. Plasma extracted from recovered COVID-19 patients is known as convalescent plasma (CP). CP contains antibodies of various diversity (polyclonal) and affinities to SARS-CoV-2 and was greatly employed during the early phases of the pandemic. More recently, monoclonal antibodies (mAbs) and nanobodies/sybodies have been developed. By isolating memory B cells from recovered patients and immunized animals or screening of antibody mRNA using phage display, highly selective candidates with high-neutralization capacity have been identified. Neutralizing responses to SARS-CoV-2 target the receptor-binding domain (RBD) of the spike (S) glycoprotein, which is required to interact with the target receptor angiotensin-converting enzyme 2 (ACE2) on host cells [42–48]. Steric hindrance of the RBD–ACE2 interaction by antibodies will block viral entry and prevent infection. It should be noted that other neutralizing epitopes, distant from the RBD, exist but are less studied [43–50].

## CONVALESCENT PLASMA

CP has been used to successfully reduce mortality in a variety of viral epidemics, including influenza, SARS and Middle East Respiratory Syndrome (MERS) [51, 52]. During the current COVID-19 pandemic, several studies have investigated CP transfusions with high nAb titres as a treatment option (see Figure 2A). Plasma is harvested from donors with total anti-spike IgG titres of >1:320 using plasmapheresis, this can then be transfused into an ABO-compatible patient [53]. Table 2 summarizes studies investigating the use of CP in COVID-19 patients.

**Table 2:** Information to show the CP treatment regimen and outcome of several COVID-19 studies

Author and study type	Dose	No. of patients	Patient severity	Administration	Patient outcomes
Li *et al* [54] Open-label randomized clinical trial	>1:640 S-RBD-specific IgG	103	Control group: 29 life-threatening, 22 severe CP group: 29 life-threatening, 23 severe	4–13 ml/kg of recipient body weight	Mortality: 15.7% CP group vs. 24% control *P* = 0.30 Clinical improvement: Severe patients 91.3% CP group vs. 68.2% control *P* = 0.03 Critically ill patients 20.7% CP group vs. 24.1% control *P* = 0.83
Duan *et al.* [55] Case series	>1:640 nAb	10	10 severe	1 dose of 200 ml	All recovered No severe adverse effects observed
Shen *et al*. [56] Case series	>1:80 nAb	5	5 critically ill	2 transfusions of 200 ml	Of the five patients, three discharged and two were in stable condition
Liu *et al.* [53] Matched control	≥1:320 S-specific IgG	39	CP group: 39 severe to life-threatening Matched controls: 152 severe to life-threatening	Two transfusions of 250 ml	12.8% mortality for CP group 24.4% mortality for matched controls (*P* = 0.039) CP improved survival in non-intubated patients (*P* = 0.015) but not for intubated patients (*P* = 0.752)
Donato *et al.* [57] Case series	>1:500 nAb	47	32 non-mechanically ventilated, 22% immunocompromised and 19% had active cancer 15 mechanically ventilated	400–500 ml	Non-mechanically ventilated: 15.6% intubation rate compared to institutional data (not reported; *P* = 0.038) 87.5% survival rate compared to 66% from institutional data (*P* = 0.012) Mechanically ventilated: 46.7% 30-day mortality rate compared to institutional data 68.5% (*P* = 0.093)
Agarwal *et al.* [58] Open-label randomized control trial	>1:20 nAb	464	Moderate illness	Two transfusions of 200ml	Progression to severe disease or mortality: 19% CP group vs. 18% control
Simonovich *et al.* [59] Double-blinded randomized control	>1:800 S-specific IgG	333	Patients with severe COVID-19 pneumonia	5–10 ml/kg of recipient body weight	Mortality: 10.96% CP group vs. 11.43% control

An early meta-analysis of CP treatment for COVID-19 found evidence of reduced mortality as well as increased viral clearance, and clinical improvements [60]. Additionally, a more recent meta-analysis of larger, better quality studies confirmed these findings [61]. However, both the PLACID and PlasmAr randomized trials found no differences in disease progression or mortality in COVID-19 patients receiving CP or best standard of care/placebo [58, 59]. Larger, blinded, randomized control trials are still ongoing to confirm the efficacy of CP treatment, the RECOVERY trial in Oxford is one such Phase 3 trial of CP (NCT04381936).

In SARS patients, early CP treatment within 14 days of infection significantly improved outcomes [62]. This has also been suggested for COVID-19, but more studies are required to fully evaluate this [55]. Recovered patients with high nAb titres have relatively stable levels but these do decrease over time. Gontu *et al*. observed that the optimal time window for recovered patients to donate plasma is within 60 d.p.s.o [9].

Finally, CP treatment could be particularly beneficial for individuals who are immunocompromised [63, 64]. The nAbs in CP are likely targeted to a range of SARS-CoV-2 S protein epitopes, which is advantageous compared to single or even ‘cocktail’ mAb treatment where there is greater likelihood of escape mutations [65].

### Monoclonal antibodies

Many studies have tested the neutralizing capacity of mAbs against SARS-CoV-2 *in vitro* (Figure 2B) and assessed their functionality *in vivo.* Neutralizing mAbs have shown a reduction in viral load and protection from challenge in animal models [42, 44–50, 66, 67]. This ability to inhibit infection highlights mAbs as potential therapeutic candidates for COVID-19.

Multiple candidates are in advanced clinical trials (Table 3). Recently, two mAb therapies (bamlanivimab, formerly LY-CoV555, and REGN-COV2) have received emergency use authorization by the Food and Drug Administration (FDA) to prevent mild-to-moderately-infected patients from progressing to severe disease. While bamlanivimab is a single mAb isolated from the B cells of a convalescent patient [68], REGN-COV2 is a cocktail of two mAbs (casirivimab and imdevimab) identified using both recovered patients and humanized mice [70]. Casirivimab and imdevimab recognize non-overlapping epitopes on the RBD which may overcome resistance posed by ‘viral escape’ mutations, such as D614G, a missense mutation in the spike protein that results in a more transmissible form of SARS-CoV-2 [72]. This approach of ‘antibody cocktails’ is also being explored by AstraZeneca, with their candidate AZD7442, comprising two mAbs, recently entering Phase 3 trials [42].

**Table 3:** Current leading mAb therapies against SARS-CoV-2 Spike/RBD in clinical trials

Company	mAb name	Comments	Stage of development	Study group
Eli Lilly and Company (Developed with AbCellera)	Bamlanivimab^a^ (LY-CoV555/LY3819253)	Human IgG1 isolated from convalesced patient using high-throughput microfluidic screening [68]	Phase 3—NCT04497987 ‘BLAZE-2’	Nursing Home residents and staff
			Phase 3—NCT04501978 ‘ACTIV-3’	Inpatients
			Phase 2/3—NCT04518410 ‘ACTIV-2’	Outpatients
			Phase 1—NCT04537910	Healthy Participants
	LY-CoV555 (LY3819253) + LY-CoV016 (LY3832479)	Combination therapy [69]	Phase 2—NCT04427501 ‘BLAZE-1’	Mild to Moderate Illness
Regeneron Pharmaceuticals	REGN-COV2^a^ (Casirivimab + Imdevimab)	Identified from humanized mice and convalescent samples. This dual-antibody cocktail target non-overlapping epitopes [70]	Phase 3—NCT04452318	Healthy adults who are household contacts with a positive case
			Phase 2/3—NCT04381936 ‘RECOVERY’	COVID-19 Patients
			Phase 1/2—NCT04425629	Ambulatory COVID-19 patients
			Phase 1/2—NCT04426695	Hospitalized patients
			Phase 1 NCT04519437	Volunteers—Healthy, Chronic stable illness
Vir Biotechnology/GlaxoSmithKline	Sotrovimab (VIR-7831/GSK4182136)	Fully human based on S309 IgG which was isolated from the memory B-cells of an individual recovered from SARS-CoV (cross-reactive) [47]	Phase 2/3— NCT04545060 ‘COMET-ICE’	Patients who are at high risk of hospitalization
AstraZeneca	AZD7442 (Tixagevimab + Cilgavimab)	Antibodies with non-overlapping epitopes identified from a convalescent patient [42]. The antibodies have been optimized to extend half-life so they should be prevalent for 6–12 months—‘Long-Acting Antibody Combination’	Phase 3—NCT04625972 ‘STORM CHASER’	Adults with potential recent (within 8 days) exposure to a confirmed positive case
			Phase 3—NCT04625725 ‘PROVENT’	Adults who have no history of SARS-CoV-2 but have been exposed
Celltrion	Regdanvimab (CT-P59)	Targets the RBD of the spike protein	Phase 2/3—NCT04602000	Diagnosed outpatients with mild conditions

Included are the most advanced candidates, determined as those that have entered Phase 2/3 clinical stage.

^a^Those which have received emergency use authorization by the FDA. Table created with aid from Yang *et al*. [71].

### Cross-reactive nAb therapies

Multiple SARS-CoV and MERS-CoV mAbs were identified following the SARS and MERS epidemics in 2003 and 2012, respectively [73]. However, therapeutic developments were limited due to the short duration of these outbreaks. Both SARS-CoV and SARS-CoV-2 utilize ACE2 as their cell-entry receptor and the S-glycoprotein of SARS-CoV-2 is over 70% identical to that of SARS-CoV [74–79]. Conversely, MERS-CoV binds to the CD26 receptor and is less homologous to SARS-CoV-2 [79, 80]. Antibody cross-reactivity could potentially allow repurposing of these SARS-CoV mAbs to combat COVID-19.

RBD-directed mAbs, which interfere with ACE2 binding, thereby neutralizing SARS-CoV (e.g. 80R, CR3014), were unable to bind to SARS-CoV-2-RBD [81, 82]. Conversely, multiple SARS-CoV-targeted mAbs, which do not compete with ACE2, have shown potent cross-neutralizing capacity including 47D11 and CR3022 [82–84]. The ability of CR3022 to neutralize SARS-CoV-2 has been disputed by Yuan *et al*., however, who used a pseudovirus neutralization assay to assess this rather than one with live virus as with Huo *et al*. [84, 85]. A further explanation for the differences seen is that antibodies that show cross-reactivity recognize epitopes that are highly conserved between the strains. For example, the epitope of CR3022 is 86% conserved between SARS-CoV and SARS-CoV-2, and the more recently identified S309 (see Table 3) binds an epitope that is 77% conserved [47, 85]. Additional work has shown that further increasing the conservation of CR3022’s epitope vastly increases the antibody’s affinity to SARS-CoV-2 RBD, suggesting that antibody cross-reactivity is highly dependent on epitope recognition [86].

### Nanobodies

Efforts have also been directed towards the development of nanobodies to treat COVID-19 (Figure 2C). Sequences of these single-domain antibodies (VHH) capable of blocking the RBD/ACE2 interaction and neutralize SARS-CoV-2 have been identified using synthetic libraries (synthetic nanobodies, sybodies) and camelids (nanobodies), which produce heavy-chain-only antibodies [13, 87–95]. Nanobodies have multiple benefits over conventional antibodies such as their biophysical and biochemical characteristics, and ease of manufacture and varied administrative potential (e.g. via inhalation) [91, 96].

Recent literature has shown a variety of ways in which antibodies can be used as treatment for COVID-19. While CP may work as a polyclonal approach, mAbs and nanobodies recognizing the RBD epitope of the virus are more promising since they are potent, high titre, relatively safe and can be readily manufactured in bulk. Because of this, multiple candidates are reaching clinical trials within a short timescale. Candidates recognizing epitopes that are highly conserved between coronaviruses have scope as potential pan-coronavirus therapies and may protect individuals from future epidemic/pandemic strains.

## ANTIBODY RESPONSES TO SARS-CoV-2 VACCINES AND LONG-TERM IMMUNITY

Prophylactic vaccines are in development to protect against COVID-19, with the aim of inducing nAb and T cell responses to combat infection. *In vivo* antiviral efficacy has been demonstrated in animal models, including preventing infection when challenged, and is being tested in clinical trials [97–117].

The majority of vaccines include the whole SARS-CoV-2 spike protein, and may also include the nucleocapsid protein (NP), while others only employ the RBD [97–109, 112–117]. The NP antigen does not generate antibodies that are neutralizing against SARS-CoV-2, whereas RBD and spike protein antigens elicit nAb responses [102]. The RBD and S1 domain of the spike protein unsurprisingly produce the greatest nAb responses, as these domains are responsible for ACE2 binding and gaining entry to host cells [118, 119]. Smith *et al.* and Yarmarkovich *et al.* took a computational approach to predict epitopes that produce humoral and cell-mediated responses, which may be broadly protective across various coronaviruses [120, 121]. Unfortunately, some non-neutralizing antibodies may have the potential to bridge viral entry into host immune cells via Fc receptors, known as antibody-dependent enhancement (ADE). This leads to increased infectivity, higher viral loads, more severe disease and has been observed in previous SARS/MERS vaccines [122]. Thus far, no study has yet shown evidence of vaccine-induced ADE for SARS-CoV-2.

The duration of long-term immunity to SARS-CoV-2 following infection or vaccination, as well as the level of nAb required for immunity, is currently unknown. Using a mathematical model of antibody kinetics determined by follow-up of coronavirus convalescent patients, one study has predicted that antibody responses will decline according to a biphasic pattern—a rapid decline initially, followed by a slower rate of decay [123]. This study indicated that, due to the substantial initial reduction of antibodies, up to 50% of patients could test seronegative after just 1 year [123]. Although these results cannot be verified until those patients are followed for several years following infection, other studies have estimated the time of seroreversion of SARS-CoV-2 antibodies based on the time taken for patients to become seronegative; 46.9 days for IgM and 51 days for IgA, as of yet, there is no consensus on IgG (Table 1) [6]. The nAb titres initially increase and remain stable for 3–4 months [5, 124–127]. Individuals with high peak nAb titres were observed to maintain these, but levels decreased to those of less severe groups at >90 d.p.s.o [5, 127].

The duration of the immune response resulting from seasonal coronavirus infection varies, but the results obtained from these can help predict the duration of antibody responses until longer-term studies with large cohorts of patients can be carried out for SARS-CoV-2. Previous work carried out on SARS-CoV has indicated convalescent patients remained IgG positive for 2–4 years and antibody responses declined after 2–3 years, with severely affected individuals more likely to maintain detectable responses [128–134]. However, antibody responses for six out of nine volunteers inoculated with seasonal coronavirus strain 229E were no longer sufficient to prevent reinfection 1 year later [135]. Furthermore, a 35-year-long study found that most seasonal coronavirus reinfections occurred every 3 years, depending on re-exposure and lingering immunity [136]. Adapted seasonal coronavirus modelling estimates that SARS-CoV-2 immunity may last approximately 45 weeks, but an antibody response may not confer complete protection from reinfection [133, 137].

Reinfection has been reported in a number of cases, summarized in Table 4. The majority of the reinfected individuals had an initial mild or asymptomatic infection, and these may not elicit a sufficiently robust antibody response to be sustained and protective since patients whose nAb responses were measured had low to undetectable responses [138–145]. These reinfection cases highlight that since most cases of COVID-19 will be mild, reinfection is possible especially following a reduction in nAbs and the possibility of spike protein mutations that reduce nAb-binding affinity [65]. Two patients were reinfected with a D614G variant, and one patient was reinfected with an N440K variant, which is a known nAb escape mutation [65, 140, 141, 143]. A recent study has demonstrated that although antibody titres decrease substantially over time, neutralization activity is retained for up to 6 months [146]. Longer studies involving more individuals are required to evaluate when people might become vulnerable to reinfection. This work supports a vaccine-based approach to controlling SARS-CoV-2 transmission but if serology of vaccinated individuals follows a similar pattern to those who have recovered, then regular boosters may be required.

**Table 4:** A summary of SARS-CoV-2 reinfection cases confirmed by whole-genome sequencing

Location	Patient: age (years) and sex (M/F)	Severity of first infection	Severity of second infection	Days between first and second infection	Reference
Hong Kong	34 (M)	Mild	Asymptomatic	142	[138]
USA	25 (M)	Mild	Severe	48	[139]
	42 (M)	Mild	Moderate	51	[140]
	60–69^a^	Severe	Mild	118	[141]
Ecuador	46 (M)	Mild	Moderate	47	[142]
India	25 (M)	Asymptomatic	Asymptomatic^b^	100	[143]
	28 (F)	Asymptomatic	Asymptomatic^b^	101	
	27 (M)	Mild	Moderate	66	[144]
	31 (M)	Asymptomatic	Mild	65	
	27 (M)	Asymptomatic	Mild	19	
	24 (F)	Mild	Moderate	55	

^a^Patient details only gave age range of 60–69 years.

^b^Asymptomatic but had a higher viral load upon reinfection.

### Conclusion

Antibodies are an important aspect of the immune response to COVID-19. While there remains a lot to learn, it is encouraging to see that in a matter of months, many promising antibody-based prophylactics and therapies are making their way into the clinic. Considering the number of reported cases of SARS-CoV-2 reinfection, the uncertainty surrounding long-term immunity will hopefully be more conclusively addressed in the months to come. To date, the current estimate of antibody longevity is 46.9 days for IgA and 51 days for IgM, with no consensus on IgG. Reinfections have occurred between 19 and 142 days, with the majority greater than 50 days, after recovery from the first infection, resulting in both mild and severe illness. These numbers could change greatly in the coming months and may not be representative of the population. It is important to stress that antibodies are not the sole immune defence against COVID-19, and many vaccines aim to elicit general adaptive immune responses. Evaluating the collective immune response to SARS-CoV-2 will advance our understanding of the mechanism of disease and its control.

## APPENDIX 1

David J. Ahern^1^, Hannah Almuttaqi^1^, Dominic S. Alonzi^2^, Aljawharah Alrubayyi^3^, Ghada Alsaleh^1^, Valentina M. T. Bart^4^, Vicky Batchelor^1^, Rebecca Bayliss^5^, Dorothée L. Berthold^1^, Jelena S. Bezbradica^1^, Tehmina Bharuchq^2^, Helene Borrmann^3^, Mariana Borsa^1^, Rowie Borst^1^, Juliane Brun^2^, Stephanie E. A. Burnell^4^, Lorenzo Capitani^4^, Athena Cavounidis^6^, Lucy Chapman^4^, Anne Chauveau^1^, Liliana Cifuentes^1^, Amy Susan Codd^4^, Ewoud Bernardus Compeer^1^, Clarissa Coveney^1^, Amy Cross^7^, Sara Danielli^1^, Luke C. Davies^4^, Calliope A. Dendrou^8^, Sandra Dimonte^4^, Ruban Rex Peter Durairaj^4^, Lynn B. Dustin^1^, Arthur Dyer^9^, Ceri Fielding^4^, Fabian Fischer^1^, Awen Gallimore^4^, Sarah Galloway^4^, Anís Gammage^1^, Ester Gea-Mallorquí^1^, Andrew Godkin^4^, Stephanie Jean Hanna^4^, Cornelia Heuberger^1^, Sarah Hulin-Curtis^4^, Fadi Issa^7^, Emma Jones^4^, Ruth Jones^10^, Kristin Ladell^4^, Sarah N. Lauder^4^, Kate Liddiard^5^, Petros Ligoxygakis^11^, Fangfang Lu^12^, Bruce MacLachlan^4^, Shayda Maleki-Toyserkani^4^, Elizabeth H. Mann^1^, Anna M. Marzeda^1^, Reginald James Matthews^13^, Julie M. Mazet^1^, Anita Milicic^14^, Emma Mitchell^4^, Owen Moon^4^, Van Dien Nguyen^4^, Miriam O'Hanlon^1^, Clara Eléonore Pavillet^15^, Dimitra Peppa^3^, Ana Pires^4^, Eleanor Pring^4^, Max Quastel^16^, Sophie Reed^4^, Jan Rehwinkel^17^, Niamh Richmond^1^, Felix Clemens Richter^1^, Alice J. B. Robinson^1^, Patrícia R. S. Rodrigues^4^, Pragati Sabberwal^4^, Arvind Sami^18^, Raphael Sanches Peres^1^, Quentin Sattentau^12^, Barbora Schonfeldova^1^, David Oliver Scourfield^4^, Tharini A. Selvakumar^16^, Freya R. Shepherd^4^, Cariad Shorten^15^, Anna Katharina Simon^1^, Adrian L. Smith^19^, Alicia Teijeira Crespo^5^, Michael Tellier^12^, Emily Thornton^17^, Lion F. K. Uhl^1^, Erinke van Grinsven^1^, Angus K. T. Wann^1^, Richard Williams^1^, Joseph D. Wilson^15^, Dingxi Zhou^1^ and Zihan Zhu^12^

### Affiliations of Consortium members

^1^Kennedy Institute of Rheumatology, Nuffield Department of Orthopaedics, Rheumatology and Musculoskeletal Sciences, University of Oxford, Oxford, UK; ^2^Oxford Glycobiology Institute, Department of Biochemistry, University of Oxford, Oxford, UK; ^3^Nuffield Department of Clinical Medicine, Nuffield Department of Medicine, University of Oxford, Oxford, UK; ^4^Division of Infection and Immunity, School of Medicine, Cardiff University, Cardiff, UK; ^5^Division of Cancer and Genetics, School of Medicine, Cardiff University, Cardiff, UK; ^6^Translational Gastroenterology Unit, Nuffield Department of Medicine, University of Oxford, Oxford, UK; ^7^Nuffield Department of Surgical Sciences, University of Oxford, Oxford, UK; ^8^Wellcome Centre for Human Genetics, Nuffield Department of Medicine, University of Oxford, Oxford, UK; ^9^Department of Oncology, University of Oxford, Oxford, UK; ^10^Dementia Research Institute, Haydn Ellis Building, Cardiff University, Cardiff, UK; ^11^Department of Biochemistry, University of Oxford, Oxford, UK; ^12^Sir William Dunn School of Pathology, Medical Science Division, University of Oxford, Oxford, UK; ^13^Centre for Medical Education, School of Medicine, Cardiff University, Cardiff, UK; ^14^The Jenner Institute, Nuffield Department of Medicine, University of Oxford, Oxford, UK; ^15^Medical Sciences Division, University of Oxford, Oxford, UK; ^16^Nuffield Department of Medicine, University of Oxford, Oxford, UK; ^17^Medical Research Council Human Immunology Unit, Medical Research Council Weatherall Institute of Molecular Medicine, Radcliffe Department of Medicine, University of Oxford, Oxford, UK; ^18^Botnar Research Centre, Nuffield Department of Orthopaedics, Rheumatology and Musculoskeletal Sciences, University of Oxford, Oxford, UK; ^19^Department of Zoology, University of Oxford, Oxford, UK
